# Major vault protein suppresses lung cancer cell proliferation by inhibiting STAT3 signaling pathway

**DOI:** 10.1186/s12885-019-5665-6

**Published:** 2019-05-15

**Authors:** Hui Bai, Chenchen Wang, Yu Qi, Jin Xu, Nan Li, Lili Chen, Bin Jiang, Xudong Zhu, Hanwen Zhang, Xiaoyu Li, Qing Yang, Junqing Ma, Yong Xu, Jingjing Ben, Qi Chen

**Affiliations:** 1 Department of Pathophysiology, Key Laboratory of Cardiovascular Disease and Molecular Intervention, Nanjing Medical University, Nanjing, China; 2 Department of Pathology, The First Affiliated Hospital of Bengbu Medical College, Bengbu Medical College, Bengbu, China; 3 Department of Molecular Cell Biology and Toxicology, Key Laboratory of Modern Toxicology, Nanjing Medical University, Nanjing, China

**Keywords:** Non-small cell lung cancer, Major vault protein, STAT3, Cell proliferation, Cell apoptosis

## Abstract

**Background:**

Major vault protein (MVP) is the major component of vault, a eukaryotic organelle involved in multiple cellular processes, and is important in multiple cellular processes and diseases including the drug resistance in cancer chemotherapies. However, the role of MVP in lung cancer remains unclear.

**Methods:**

We examined MVP expression in 120 non-small cell lung cancer (NSCLC) tumors and matched normal tissues by immunohistochemistry. Its relationship with NSCLC prognosis was determined by investigating the patient cohort and analyzing the data from a published dataset consisting with more than 1900 lung cancer patients. We further performed shRNA-introduced knockdown of MVP in Lewis lung carcinoma (LLC) cells and examined its effects on the tumor formation in a xenograft mouse model and the tumor cell proliferation, apoptosis, and signal transduction in vitro.

**Results:**

We found that MVP was up-regulated significantly in tumor tissues compared with the matched tumor-adjacent normal tissues. The increased expression of MVP in lung adenocarcinoma was associated with a better prognosis. Knockdown of MVP in LLC cells promoted xenografted lung cancer formation in mice, which was accompanied with accelerated tumor cell proliferation and suppressed cell apoptosis in vitro. Knockdown of MVP stimulated STAT3 phosphorylation, nuclear localization, and activation of JAK2 and RAF/MEK/ERK pathways in LLC cells. Administration of STAT3 inhibitor WP1066 could prevent MVP knockdown induced tumorigenesis.

**Conclusions:**

Our findings demonstrate that MVP may act as a lung tumor suppressor via inhibiting STAT3 pathway. MVP would be a potential target for novel therapies of lung adenocarcinoma.

**Electronic supplementary material:**

The online version of this article (10.1186/s12885-019-5665-6) contains supplementary material, which is available to authorized users.

## Background

Lung cancer is one of the most common cancers and the leading cause of cancer related death [1]. Non-small cell lung cancer (NSCLC) accounts for about 80% of diagnosed patients with lung cancer [2]. Despite the progress on targeted therapies [3, 4], understanding the complicated pathogenesis mechanisms is still limited. Moreover, rapid progression on drug resistance affects the effectiveness of chemotherapies and targeted therapies. Thus, identification of novel biomarkers and targets are useful in developing new therapeutic options for NSCLC.

Major vault protein (MVP), also known as lung resistant protein (LRP), is ubiquitously expressed in most animal cells. It is the major component of vault that is the largest known ribonucleoprotein particle in cytoplasm [5]. Current studies indicate that vault is involved in a broad range of cellular processes, including nuclear pore assembly, subcellular transportation, cell signaling, and interferon response [5–8]. Although MVP is overexpressed in the drug-resistant cancer cells [9–11], the definite role of it in NSCLC is still a disputable issue [12, 13]. Janikova et al. reported that the MVP expression is of prognostic significance in NSCLC when examined in combination with miR-23b [14]. MVP is required for the nuclear localization of tumor suppressor PTEN [15, 16], which down-regulates cyclin D1, prevents the phosphorylation of MAPK, and leads to cell cycle arrest [17]. MVP also binds to HIF1α and promotes the degradation of HIF1α. It supports the notion that MVP may function as a tumor suppressor in renal adenocarcinoma cells [18]. Yet, MVP also promotes survival and migration of glioblastoma [19], and suppresses apoptosis of human senescent diploid fibroblasts [20] and human colon cancer cells [21]. These inconsistent results suggest that insighted mechanistic researches on the role of MVP in cancer are absolutely necessary.

By examining 120 patients with NSCLC, we found that MVP expression was significantly up-regulated in cancer tissues compared with the paired normal tissues. Higher MVP expression was correlated with better clinical outcomes in patients with lung cancer, especially in patients with adenocarcinoma. When MVP was knocked down in Lewis lung carcinoma (LLC) cells and the MVP suppressed LLC cells were injected subcutaneously into mice, it promoted lung cancer growth and the tumor cell apoptosis was inhibited. This was causally linked to the activation of STAT3 signaling pathway. Our findings suggest that MVP act as a NSCLC suppressor which may be useful for discovery of novel therapy.

## Methods

### Patient cohort and tissue collection

This study was approved by the Institution Review Board of Bengbu Medical College and Nanjing Medical University. Patients were recruited from 2011 to 2013 in the First Affiliated Hospital of Bengbu Medical College. The cohort is composed of 44 patients with adenocarcinoma and 76 patients with squamous cell carcinomas without previous lung cancer history or preoperative chemotherapy and radiotherapy.

The lung cancer samples and matched noncancerous lung tissues (more than 5 cm from the tumoral margins) were applied for the tissue microarray (TMA) construction. The TMAs were created by contract service at Shanghai OUTDO Biotech, China. Duplicate 1.0-mm diameter cores of tissue from each sample were punched from paraffin tumor block and corresponding nontumoral tissues. As a tissue control, the biopsies of normal lung tissues were inserted in the angles of each slide.

### Immunohistochemistry

The MVP antibody (1:100 dilution, Santa Cruz) was used to determine the protein expression levels. The goat IgG served as a negative control. The immunoreactivity score (IRS) were evaluated by two pathologists independently using the following semiquantitative criterion: the intensity of immunostaining was recorded as 0–3 (0, negative; 1, weak; 2, moderate; 3, strong); the percentage of immunoreactive cells was recorded as 1–4 (1, 0–25%; 2, 26–50%; 3, 51–75%; 4, 76–100%). The IRS was calculated by multiple the intensity and percentage of immunoreactive cells. Wilcoxon test (raw scores) was applied to determine the significance of MVP staining in primary lung tumors compared with the matched adjacent tumoral tissues. Mouse tumor tissues were formalin-fixed, paraffin-embedded, and sectioned. Immunohistochemistry (IHC) of mouse tissue sections was conducted with antibodies against Ki67 and CD31 (Abcam).

### Animal experiments

All aspects of the animal care and experimental protocols were approved by Nanjing Medical University Committee on Animal Care. The C57BL/6 J mice were purchased from Animal Core Facility of Nanjing Medical University and kept in animal care facilities under pathogen-free conditions. Tumor xenograft assays were performed with 6 to 8-week-old mice. Briefly, 5 × 10^6^ tumor cells per site were suspended in 0.1 ml PBS and subcutaneous injected into mice. After 3 weeks, mice were euthanized by carbon dioxide, and tumors were dissected for determining the size and weight, followed by IHC staining and flow cytometry analysis. Tumor volume was calculated using the formula: volume = 0.5236 × length × width^2^.

### Cell culture

The mouse Lewis lung carcinoma (LLC) and human lung adenocarcinoma SPC-A1 cells (Chinese Academy of Science) were cultured in DMEM medium containing 10% FBS with the supply of 5% CO_2_. To generate stable knockdown cells, the lenti-viruses including hairpin (Genepharma, China) were used to infect LLC cells. After infecting for 24 h, cells were selected by 8 μg/ml puromycin for at least 2 weeks before conducting experiments. The hairpins sequences against mouse MVP were: shRNA-MVP1, CATAAGAACTCAGCACGTATTCAAGAGATACGTGCTGAGTTCTTATG; shRNA-MVP2, CCATCGAAACTGCAGATCATTCAAGAGATGATCTGCAGTTTCGATGG. The hairpin sequence against human MVP was: shRNA-hMVP, GGTGCTGTTTGATGTCACATTCAAGAGATGTGACATCAAACAGCACC.

### Cell proliferation and apoptosis analyses

Cell proliferation assays were evaluated by the cell counting kit-8 assay (CCK-8) (Dojindo Laboratories, Kumamoto, Japan). Briefly, 2 × 10^3^ cells were seeded in 96-well plates and cultured for 1 to 6 days. Then, the CCK-8 assays were conducted according to the instruction followed by 450 nm absorbance measurement using a plate reader. For colony formation assays, cells were seeded in 10 cm-plates at a density of 200 cells per plate. After culturing for 14 days, cells were fixed with 4% paraformaldehyde for 30 min and stained with 0.1% crystal violet for 20 min. Three randomly selected areas were used to count the number of colonies. For Edu assays, cells were seeded into 96-well plates at a density of 3 × 10^3^ cells per well. Cells were treated with serum-free medium for 24 h, regular medium for 24 h, and regular medium plus the Edu for 2 h. Cells were then fixed with 4% PFA and stained with DAPI.

### Flow cytometry analysis

Cells were harvested in logarithmic growth phase, washed with PBS and fixed in 70% ethanol at 4 °C for at least 12 h. Then, the cells were washed in cold PBS, stained with propidium iodide (PI) in the darkness for 30 min and resuspended in PBS at 4 °C before analyzed by BD FACS Calibur. Annexin V/PI apoptosis detection kits (BD Biosciences) were used to detect apoptotic cells. Flow cytometry data were analyzed by using BD CELLQUEST software supplied with the instrument. Flow cytometry for the LLC tumor stromal cells was conducted as previously described [22]. Briefly, flow cytometric identification of the cells was performed through labeling with FITC-labeled CD11b antibody (AbD Serotec), APC-labeled Gr1 antibody (BD Biosciences), PE-labeled F4/80 antibody (R&D System) and APC-labeled CD11c antibody (eBioscience) or FITC-labled CD206 antibody (AbD Serotec).

### Western blot

Western blot was conducted as previously described [23]. Primary antibodies against Cyclin-D, p-Rb, Rb, Capase 3, p-STAT3, STAT3, LMNB1, p-JAK, JAK, p-Raf, Raf, p-MEK, MEK, p-ERK, ERK, p-AKT, and AKT were purchased from Cell Signaling Technology. Antibodies against MVP and GAPDH were purchased from Santa Cruz Biotechnology. Quantification was performed with Image J.

### Luciferase reporter assay

LLC cells that were grown to 80% confluence in 24-well plates were co-transfected with the pGL6-STAT3-luciferase reporter plasmid and pRL-TK plasmid (Promega) at an appropriate ratio using Lipofectamine 2000 (Invitrogen). Luciferase activity was assayed after 24 h using the dual-reporter luciferase system on a GloMax-96 luminometer (Promega).

### Statistical analysis

Statistical analysis was performed using Stata 15.0 software. For experimental data, continuous values were described with Mean ± standard error (SE) and tested by ANOVA followed by Bonferroni correction. For the patient cohort, the median value was regarded as cut-off value. Unpaired Student’s *t* test was done for the comparison between two groups. Wilcoxon test (raw scores) was applied to determine the significance of MVP staining in primary lung tumors compared with the matched adjacent tumoral tissues. Kaplan-Meier survival analysis was performed and tested by Log-rank test. The association between the transcriptional levels of MVP and overall survival of NSCLC patients was evaluated by an online database (http://kmplot.com/analysis/). The analysis parameters were set as following: split patients by “Auto select best cutoff”, probe set options using “only JetSet best probe set”, array quality control “exclude biased arrays”. Error bars represent SE for all figures. Statistical significance was defined as follows: *, *P* < 0.05; **, *P* < 0.01.

## Results

### MVP expression is increased in NSCLC associating with better clinical outcomes

**Fig. 1:**
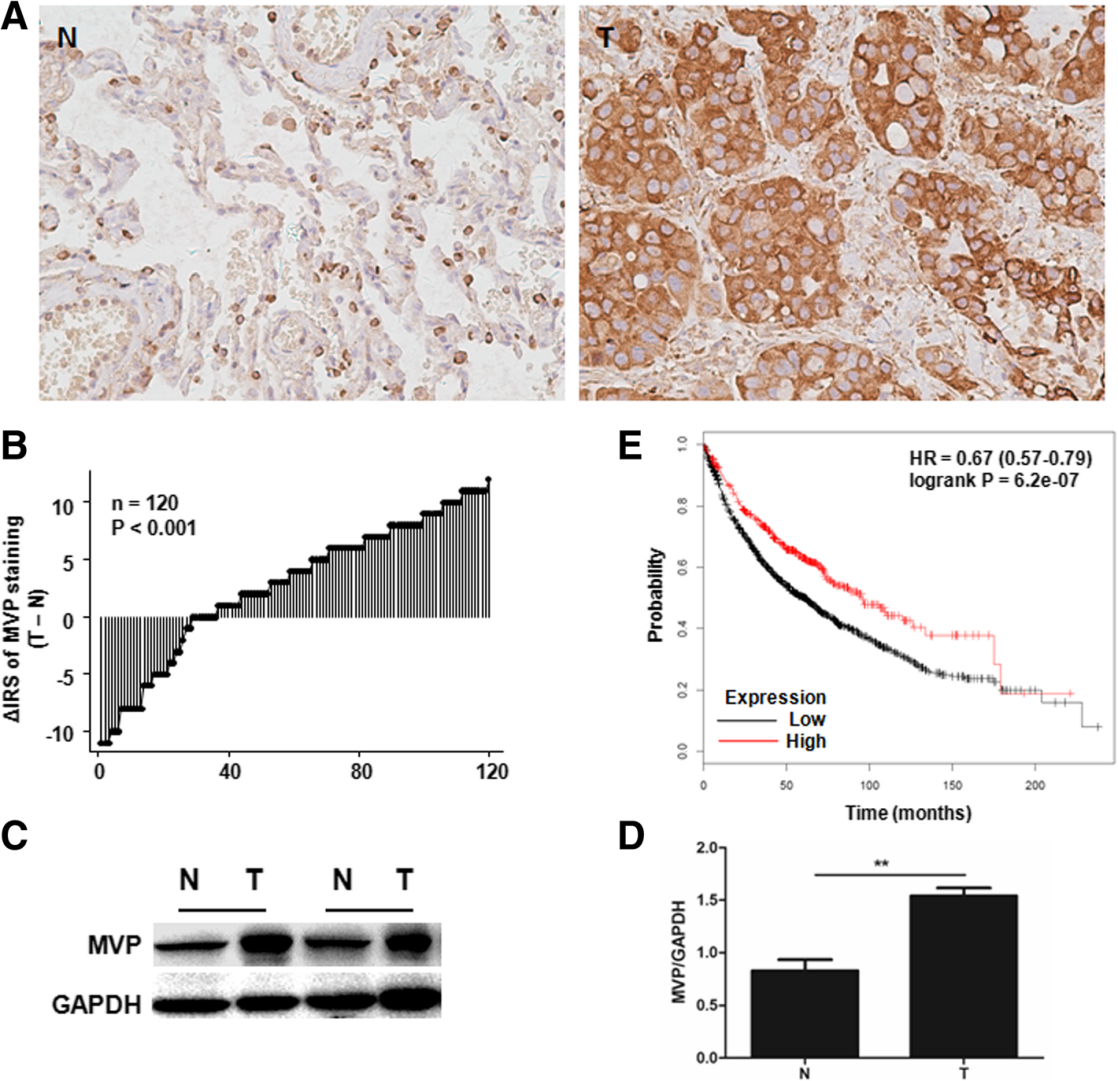
Expression of MVP in human tumor and tumor-adjacent normal tissues of NSCLC. **a.** Representative IHC staining of MVP in tumor (T) and tumor-adjacent normal (N) tissues. **b.** Distribution of the difference in MVP staining in lung cancerous tissues compared with corresponding normal tissues. IRS: immunoreactivity score. **c.** Western blot analysis of MVP in 2 pairs of randomly selected lung cancerous tissues (T) and tumor-adjacent normal (N) tissues. **d.** Quantification of MVP expression in 15 pairs of lung cancerous tissues (T) and tumor-adjacent normal (N) tissues based on western blot analysis. ** *P* < 0.01. **e.** Kaplan-Meier plot of lung cancer patients with high or low expression of MVP

To investigate the role of MVP in NSCLC, we examined MVP expression in surgically removed tumors from 120 patients with lung cancer by IHC. The detailed clinicopathological parameters are depicted in Table 1. MVP expression was significantly increased in the tumor tissues compared to the tumor-adjacent normal tissues. Tumoral MVP expression was significantly increased, compared to their normal counterparts, in 84 of 120 (70.0%) patients (Fig. 1a, b). A significant difference in MVP staining pattern was observed between the two groups (*P* < 0.001, Wilcoxon test). We also randomly selected 15 pairs of the tumor and the tumor-adjacent normal tissues, which were from 12 patients with adenocarcinoma and 3 patients with squamous cell carcinoma that had not received any preoperative chemotherapy or radiotherapy, for western blot analysis. Consistent results were obtained in Fig. 1c and d. To explore the biological significance of MVP in lung cancer progression, we conducted Kaplan-Meier estimation with published lung cancer datasets [24]. Totally 1926 patients were included in the analysis, providing strong statistic power. Patients with higher MVP expression had better clinical outcomes (HR = 0.67, 95% CI: 0.57–0.79, *P* = 6.2e-07) (Fig. 1e). The patients were stratified according to their pathology. As shown in Additional file 1: Figure S1A and B, MVP significantly increased the overall survival time of patients with adenocarcinoma but which was not detected in patients with squamous cell carcinoma. Patients not received chemotherapy or radiotherapy but with higher MVP expression had a trend of better outcome, though the difference was not statistical (*P* = 0.075) (Additional file 1: Figure S1C). In patients received both chemotherapy and radiotherapy, higher MVP expression indicated better prognosis (*P* = 0.0065) (Additional file 1: Figure S1D). Furthermore, we investigated the Kaplan-Meier curve in our own established cohort. Consistently, adenocarcinoma patients with higher MVP expression had better prognosis and less lymph node metastasis (Additional file 1: Figure S1F, Table 1). Taken together, these results suggest that MVP be associated with the pathogenesis of lung cancer, especially with adenocarcinoma.

**Table 1:** The expression of MVP and clinicopathologic parameters of patients with lung cancer

	All patients (*n* = 120)				Patients with adenocarcinoma (*n* = 44)				Patients with squamous cell carcinoma (*n* = 76)			
	Sum. (%)	Low(%)	High(%)	*P*	Sum.(%)	Low(%)	High(%)	*P*	Sum. (%)	Low (%)	High (%)	*P*
Age (years)	59.4 ± 8.4	59.7 ± 7.9	58.8 ± 9.4	0.55^*a*^	57.6 ± 7.9	56.9 ± 7.8	58.8 ± 8.0	0.44^*a*^	60.4 ± 8.7	61.4 ± 7.5	58.8 ± 10.2	0.19^*a*^
Gender				0.66^*b*^				1.00^*b*^				0.24^*b*^
Male	92(76.7)	56(60.9)	36(39.1)		23(52.3)	15(65.2)	8(34.8)		69(90.8)	41(59.4)	28(40.6)	
Female	28(23.3)	19(67.9)	9(32.1)		21(47.7)	13(61.9)	8(38.1)		7(9.0)	6(85.7)	1(14.3)	
Depth of invasion				0.81^*b*^				0.64^*b*^				0.56^*b*^
T1/T2	100(83.3)	63(63.0)	37(37.0)		39(88.6)	24(61.5)	15(38.5)		61(80.3)	39(63.9)	22(36.1)	
T3	20(16.7)	12(60.0)	8(40.0)		5(11.4)	4(80.0)	1(20.0)		15(19.7)	8(53.3)	7(46.7)	
Lymph node metastasis				0.45^*b*^				0.01^*b*^				0.48^*b*^
N0	60(50.0)	35(58.3)	25(41.7)		24(54.5)	11(45.8)	13(54.2)		36(47.4)	24(66.7)	12(33.3)	
N1/N2/N3	60(50.0)	40(66.7)	20(33.3)		20(45.5)	17(85.0)	3(15.0)		40(52.6)	23(57.5)	17(42.5)	
Distant metastasis				1.00^*b*^				1.00^*b*^				–
M0	119(99.0)	74(62.2)	45(37.8)		43(97.7)	27(62.8)	16(37.2)		76(100.0)	47(61.8)	29(38.2)	
M1	1(0.8)	1(100.0)	0(0.0)		1(2.3)	1(100.0)	0(0.0)		0(0.0)	0(0.0)	0(0.0)	
TNM stage				0.19^*b*^				0.09^*b*^				0.94^*b*^
I	40(33.3)	23(57.5)	17(42.5)		16(36.4)	8(50.0)	8(50.0)		24(31.6)	15(62.5)	9(37.5)	
II	65(54.2)	39(60.0)	26(40.0)		20(45.5)	12(60.0)	8(40.0)		45(59.2)	27(60.0)	18(40.0)	
III	14(11.7)	12(85.7)	2(14.3)		7(15.8)	7(100.0)	0(0.0)		7(9.2)	5(71.4)	2(28.6)	
IV	1(0.8)	1(100.0)	0(0.0)		1(2.3)	1(100.0)	0(0.0)		0(0.0)	0(0.0)	0(0.0)	
Tumor diameter	4.7 ± 2.2	4.7 ± 2.3	4.6 ± 2.1	0.73 ^*a*^	4.0 ± 2.0	4.1 ± 2.0	3.7 ± 2.0	0.54 ^*a*^	5.0 ± 2.2	5.1 ± 2.4	5.0 ± 2.1	0.94 ^*a*^
Pathology				1.00^*b*^				–				–
Adenocarcinoma	44(36.7)	28(63.6)	16(36.4)		–	–	–		–	–	–	
Squamous cell carcinoma	76(63.3)	47(61.8)	29(38.2)		–	–	–		–	–	–	
Mediam survival time (month)	32	31	36	0.26 ^*c*^	32	25	47	0.04 ^*c*^	32	35	30	0.84 ^*c*^

^*a*^Independent sample *t* test

^*b*^Fisher exact probability test

^*c*^Logrank test

### MVP knockdown promotes LLC tumor growth in mice

**Fig. 2:**
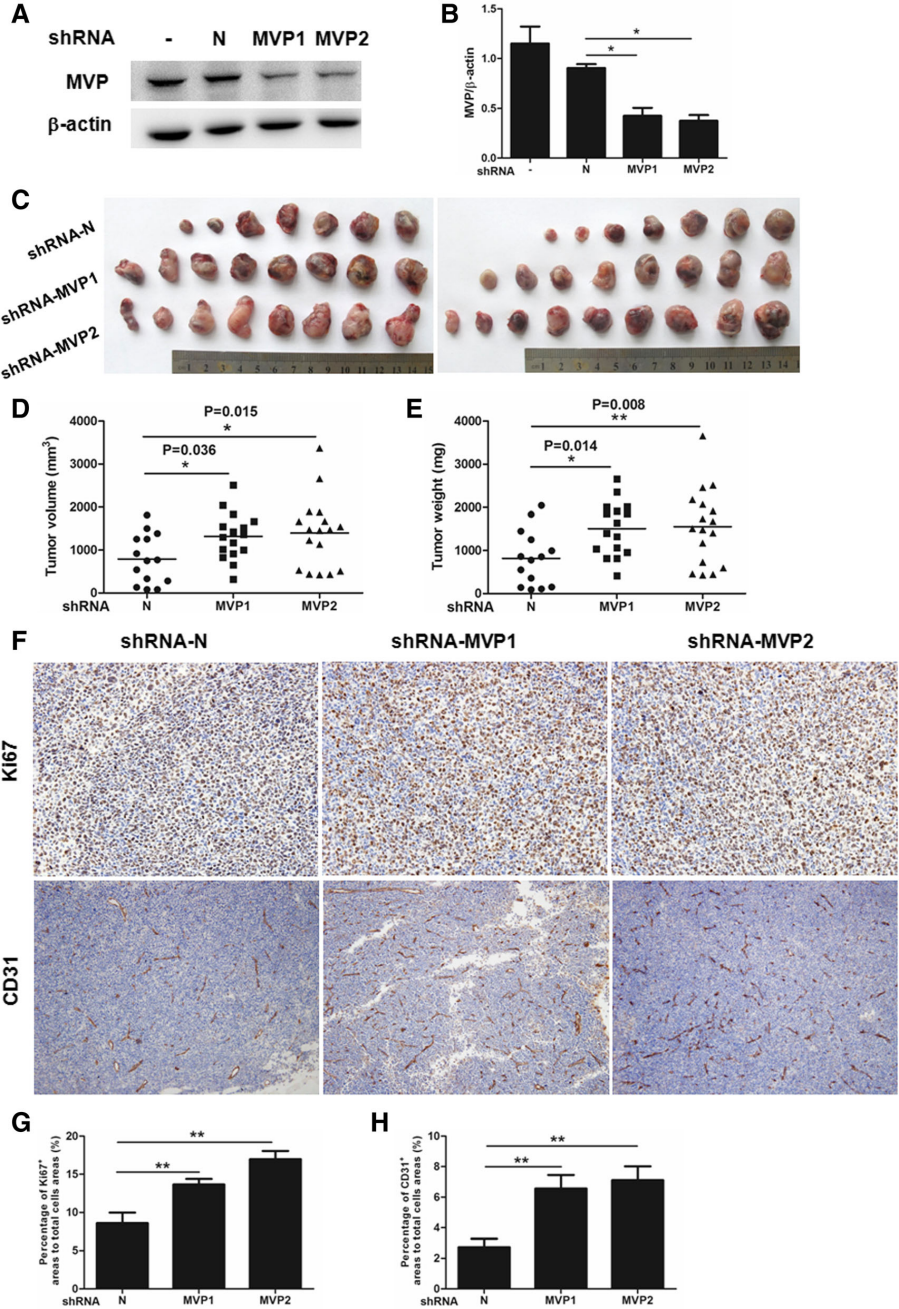
Knockdown of MVP accelerates xenografted LLC tumor growth in mice. LLC cells were infected by the lenti-shRNA against MVP for 24 h. After selection by puromycin for at least 2 weeks, the stably transfected LLC cells were subcutaneously injected into C57BL/6 mice. Three weeks later the tumor tissues were isolated for measurements. **a** Western blot analysis of MVP in the transfected LLC cells. **b** Quantification of MVP expression in the transfected LLC cells. (*n* = 3, * *P* < 0.05). **c** Images of xenografted tumors isolated from the mice. **d, e** Quantification of volume and weight of the xenografted tumors. (shRNA-N, *n* = 14; shRNA-MVP1, *n* = 16; shRNA-MVP2, *n* = 17, * *P* < 0.05) **f.** Representative IHC staining of Ki67 and CD31 in mouse tumor tissues. **g, h** Quantification of the IHC staining (*n* = 7, ** *P* < 0.05)

To determine the exact role of MVP in lung cancer, two independent hairpins against MVP and a control hairpin were respectively transfected into LLC cells to generate stable cell lines. Western blot showed significant decrease in MVP expression in both testing hairpins transfected cells compared with the untreated cells or cells transfected by control hairpin (Fig. 2a and b). We then injected subcutaneously the lenti-shRNA stably transfected LLC cells into C57BL/6 mice to monitor tumor growth in vivo. Three weeks after injection, tumor tissues were isolated for measurements. We found that tumors carrying MVP hairpins were significantly larger and heavier than the tumors carrying control hairpin (Fig. 2c-e). Consistently, IHC analysis indicated that Ki67 and CD31, a cell division marker and an angiogenesis marker respectively, were enhanced in both MVP knockdown groups (Fig. 2f-h). There were no obvious changes in tumor-associated macrophages accumulation, polarization and neutrophils recruitment in MVP knockdown tumors (Additional file 2: Figure S2). These data reveal that knockdown of MVP in tumor cells may promote lung cancer growth in mice.

### MVP knockdown promotes proliferation of tumor cells

**Fig. 3:**
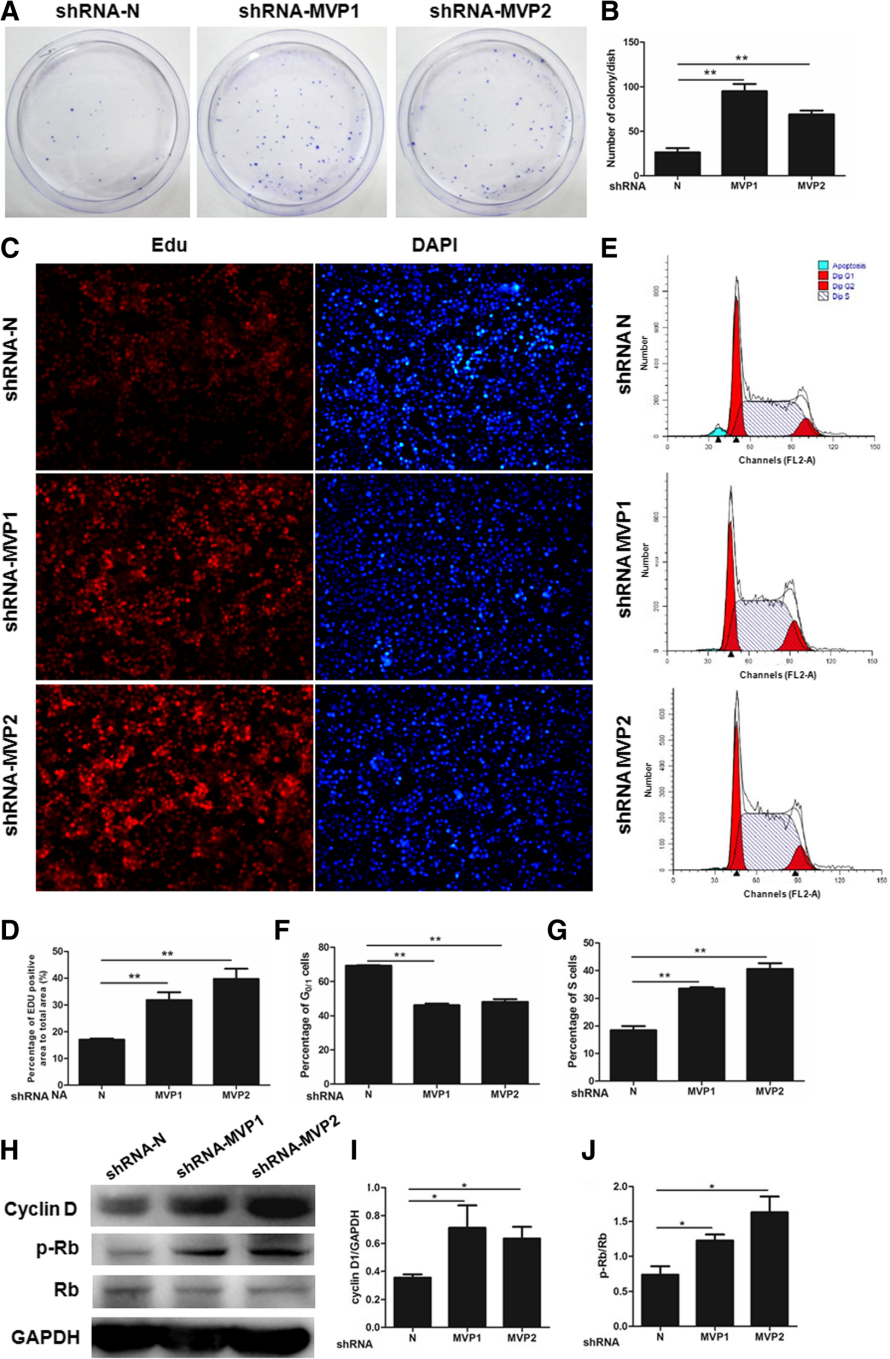
Knockdown of MVP promotes LLC cell proliferation. **a** Representative images of colony formation of the transfected LLC cells. Cells were seeded and cultured for 14 days. After fixed and stained, the cell colonies were counted. **b** Quantification of the colony formation. (*n* = 3, ** *P* < 0.01). **c.** Representative immunofluorescence staining of Edu and DAPI in the transfected LLC cells. Cells were treated with serum-free medium for 24 h, regular medium for 24 h, and regular medium plus the Edu for 2 h. Cells were then fixed with 4% PFA and stained with DAPI. **d** Quantification of the Edu staining. (*n* = 3, ** *P* < 0.01). **e** Cell cycle profiles of the lenti-shRNA stablely transfected LLC cells. Cells were harvested, washed with PBS and fixed in 70% ethanol at 4 °C for at least 12 h. After washed in PBS, stained with PI for 30 min and resuspended in PBS, cells were measured by a flow cytometry. **f, g** Quantification of the cell cycle profiles. (*n* = 3, ** *P* < 0.01). **h** Representative western blot of the indicated proteins in the transfected LLC cells. **i, j** Quantification of the western blot. (*n* = 3, * *P* < 0.05)

In order to verify if the accelerated lung cancer growth was due to stronger tumor cell proliferation, we performed colony formation assays to determine the role of MVP in cell proliferation. As shown in Fig. 3a and b, there were few clones of LLC cells carrying control hairpin. But MVP-suppressed cells formed much more clones. Similar results were found in human lung adenocarcinoma SPC-A1 cells (Additional file 3: Figure S3A and B). There were more Edu signals in LLC cells transfected by hairpins against MVP, indicating an accelerated cell division in cells (Fig. 3c, d). Next, we profiled cell cycle in LLC cells by propidium iodide (PI) staining followed by flow cytometry analysis. A significant sub-G1 population was observed in control LLC cells (Fig. 3e), indicating that a subgroup of cells underwent apoptosis. This sub-G1 population disappeared in both MVP knockdown cells. Moreover, knockdown of MVP led to an increase in S phase cells and a decrease in G1/0 phase cells (Fig. 3e-g). MVP knockdown also promoted the proliferation of SPC-A1 cells and inhibited the apoptosis (Additional file 3: Figure S3C and D).

We further examined changes in cell cycle modulators. Knockdown of MVP led to an up-regulation of Cyclin D in LLC cells (Fig. 3h, i). Coordinately, enhanced phosphorylation of tumor suppressor Rb (p-Rb), an inactive form of Rb and a product of Cyclin D-associated CDKs, was found in the MVP suppressed cells (Fig. 3h, j). Collectively, our results demonstrate that MVP may be a negative regulator on lung cell proliferation.

### MVP knockdown inhibits apoptosis of tumor cells

**Fig. 4:**
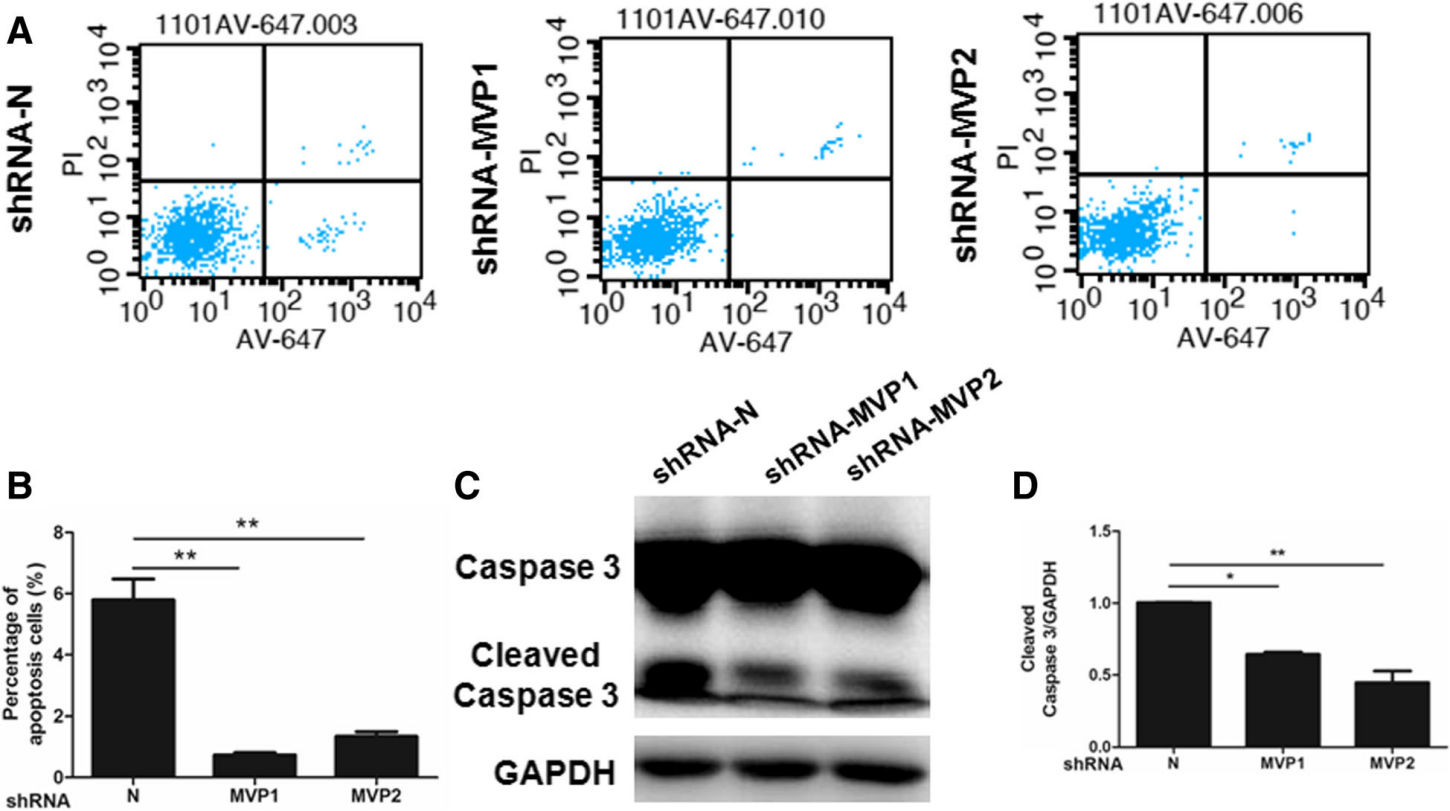
Knockdown of MVP inhibits LLC cell apoptosis. **a** Representative flow cytometry of the transfected LLC cells with PI and Annexin V-647 staining. **b** Quantification of the flow cytometry. (*n* = 4, ** *P* < 0.01). **c** Representative western blot of the indicated proteins in the transfected LLC cells. **d** Quantification of the western blot. (*n* = 3, * *P* < 0.05, ** *P* < 0.01)

Since we had observed a decrease of sub-G1 population in MVP knockdown cells, we further investigated the effects of MVP on apoptosis. The flow cytometry assays showed a significant population (about 6%) of LLC cells undergoing apoptosis. Knockdown of MVP dramatically decreased the percentage of apoptotic cells (Fig. 4a, b). Similar results were also found in SPC-A1 cells (Additional file 3: Figure S3D). Because Caspase-3 is the convergence point for different signaling pathway branches in apoptosis, cleaved Caspase-3 has been widely used as an active marker of apoptosis. We measured Caspase-3 cleavage in LLC cells and found that cleaved Caspase-3 was dramatically decreased in MVP suppressed cells (Fig. 4c, d). As such, MVP may inhibit apoptosis of lung cancer cells.

### MVP knockdown activates JAK2, ERK, and STAT3 signal pathways

**Fig. 5:**
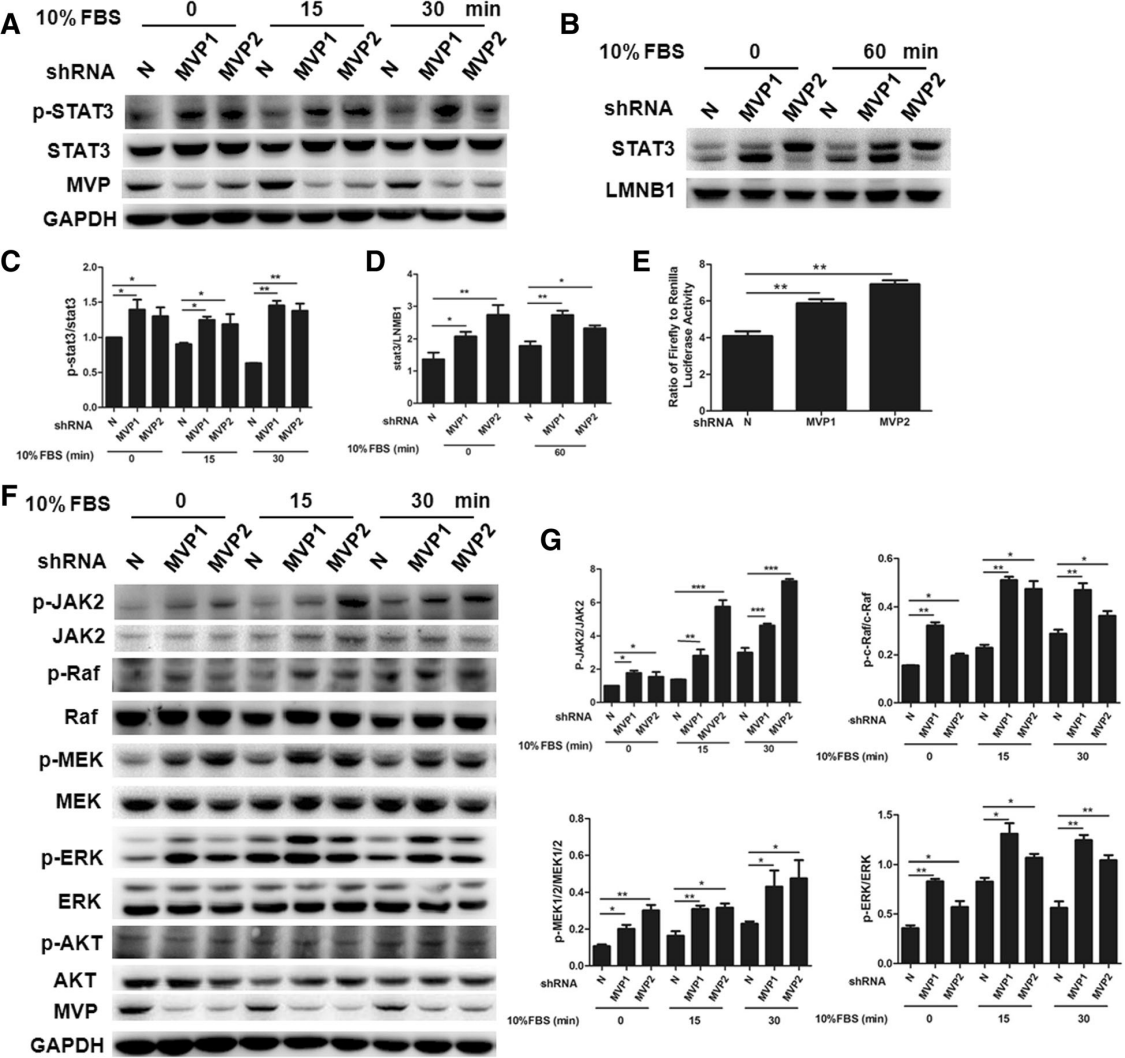
MVP knockdown activates STAT3, JAK2, and ERK in the transfected LLC cells. **a** Representative western blot of the indicated proteins in the transfected LLC cells after serum starvation and stimulation. **b** Representative western blot of nuclear STAT3 in the transfected LLC cells. **c, d** Quantification of the western blot in A and B. (*n* = 3, * *P* < 0.05, ** *P* < 0.01). **e** Luciferase reporter assay of STAT3 transcriptional activity in the transfected LLC cells. **f** Representative western blot of the indicated proteins in the transfected LLC cells after serum starvation and stimulation. **g** Quantification of the western blot. (*n* = 3, * *P* < 0.05, ** *P* < 0.01)

As a signal transducer and transcription factor, STAT3 is frequently activated in cancer cells and associated with cell proliferation, inhibition of apoptosis, and tumor progression [25, 26]. We analyzed STAT3 activity in tumor cells to understand mechanisms underlying the anti-tumor activity of MVP. When FBS was used for the activation of starved tumor cells, we found that STAT3 phosphorylation was enhanced in the MVP knockdown LLC cells in both basal level group and FBS stimulated group (Fig. 5a, c). Additionally, knockdown of MVP increased nuclear localization of STAT3 in cells (Fig. 5b, d). We further determined STAT3 transcriptional activity by performing luciferase reporter assays. When luciferase reporter controlled by a STAT3-dependent promoter was introduced into LCC cells, the luciferase activity was significantly higher in the MVP knockdown cells than in the control cells (Fig. 5e), indicating the increased STAT3 transcriptional activity after knockdown of MVP.

STAT3 is activated by receptor-associated tyrosine kinases JAKs [27]. MAPK/ERK pathway [28] and PI3K-Akt pathway [29] also regulate STAT3 activity. To determine the dominate regulator of STAT3 in the MVP knockdown cells, we determined the phosphorylation of major transducers in all three pathways. We found that knockdown of MVP enhanced phosphorylation of JAK2 and ERK at both basal level and after serum addition (Fig. 5f, g). On the contrast, no significant difference on phosphorylation of AKT was observed (Fig. 5f). These results indicate that JAK2 and ERK pathways but not AKT pathway were involved in the MVP regulated STAT3 phosphorylation. Consistently, enhanced phosphorylation of other signal molecules in MAPK/ERK, such as RAF and MEK, were detected in the MVP knockdown cells (Fig. 5f, g).

### STAT3 is required for MVP knockdown induced lung cancer cell growth

**Fig. 6:**
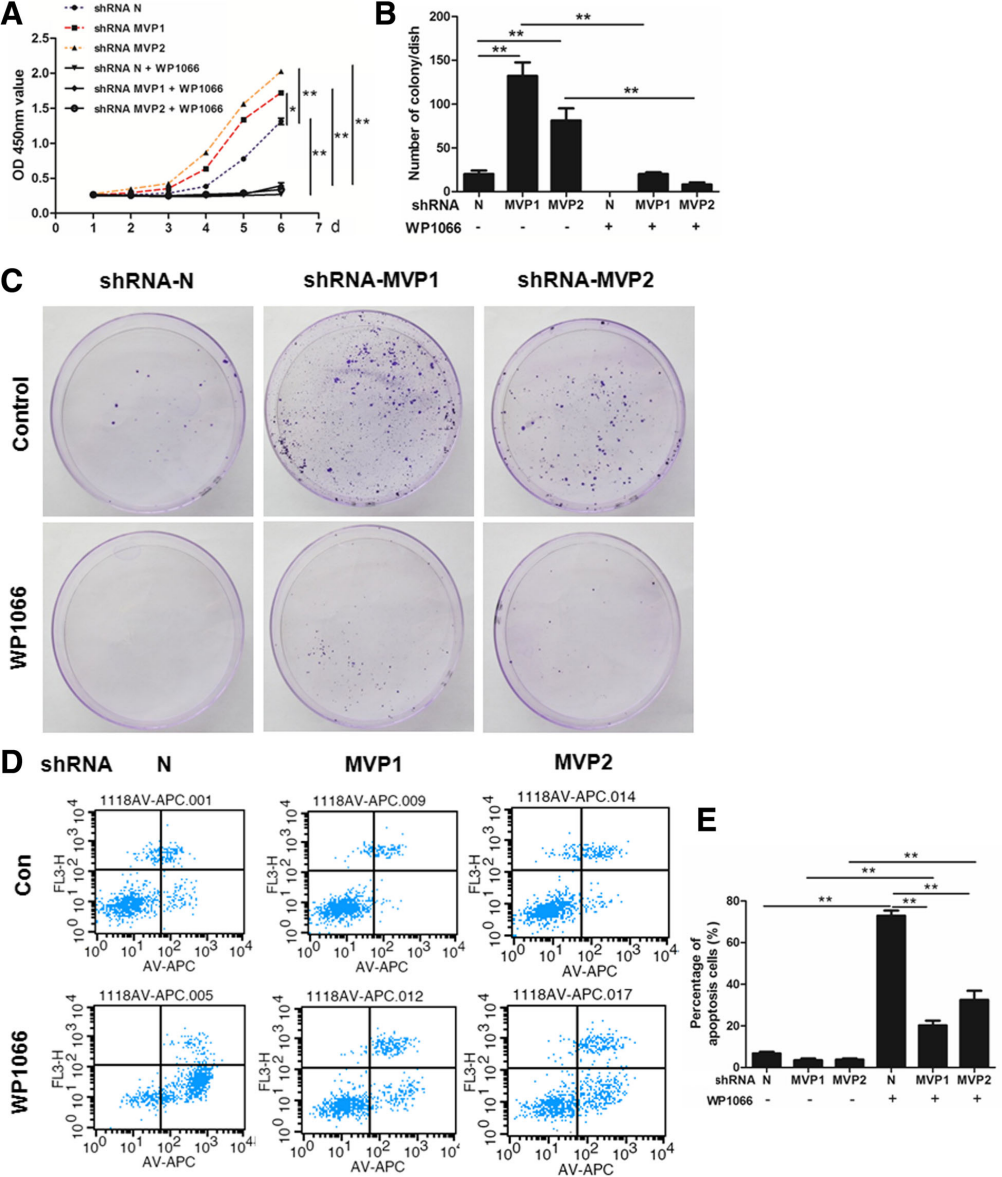
STAT3 is requisite for the effect of MVP in the transfected LLC cells. **a** CCK8 assays for the proliferation of the transfected LLC cells. (*n* = 6. * *P* < 0.05, ** *P* < 0.01) **b** and **c.** Measurements for the colony formation of the transfected LLC cells. (*n* = 3, ** *P* < 0.01). **d** Representative flow cytometry for the apoptosis of the transfected LLC cells. **e** Quantification of the flow cytometry. (*n* = 3, ** *P* < 0.01)

We had shown that MVP knockdown activated STAT3 in LLC cells. To further determined the role of STAT3 in MVP related anti-tumorigenesis, a STAT3 inhibitor WP1066 was used in the study. We found that MVP knockdown induced acceleration in LLC cell proliferation was obviously dampened by treatment with WP1066. There was no significant difference between control group and MVP knockdown groups when cells were treated by WP1066 (Fig. 6a). Similarly, WP1066 treatment could rescue MVP knockdown induced increase in colony formation (Fig. 6b, c). Furthermore, WP1066 also increased LLC cell apoptosis in MVP knockdown cells (Fig. 6d, e). Therefore, these results suggested that STAT3 activity be requisite for the tumorigenesis caused by MVP knockdown.

## Discussion

Lung cancer, causing more deaths than any other cancer types, accounts for approximately one fourth of all cancer-related deaths [1]. Treatment of lung cancer is of great importance clinically and new approaches on targeted therapies by molecular subtyping have been developed [2]. For example, Gefitinib and other EGFR tyrosine kinase inhibitors (TKIs) have been developed to treat patients with EGFR mutations which may be the most successful targeted therapy [30]. ALK inhibitor Crizotinib has been approved for treatment of patients carrying EML4-ALK fusion mutation [4]. Therapies targeting K-RAS mutation, MET amplification, ROS1 rearrangements, and other oncogenic drivers are under clinical trials [31]. However, only 10 to 25% NSCLC patients carry EGFR mutations [31]. Other drivers have ever smaller incidences. Therefore, identifying novel target genes for lung cancer therapy is urgent. In the present study, MVP was found to be of anti-tumor property with potential application for treatment of NSCLC.

It is intriguing that MVP expression was increased in surgical removed NSCLC compared with paired normal-adjacent tissues. Similar reaction pattern of MVP is also found in pancreatic tumor [32]. The up-regulation of MVP is likely triggered by the increased transcription activity in tumor cells. The core promoter sequence of MVP contains several putative transcription factor binding sites for p53 and YB-1, which are increased in tumorigenesis [32–34]. The up-regulation of tumor suppressor like p53 may constitute a feedback response in which transformed cells manage to restore normal growth controls. Malignant lesions are probably the consequences of escaping or overcoming the MVP-associated anti-tumor regulations. Indeed, our in vivo and in vitro studies reveal that suppression of MVP in lung cancer cells resulted in robust tumor growth and suppressive tumor cell apoptosis. Interestingly, the up-regulation of MVP has also been found in other situations including chemotherapy resistance, malignant transformation, as well as exposure to diverse antineoplastic drugs [5], suggesting the complexity in regulation of MVP expression in tumor cells. Yet, our population investigation reveals a better clinical outcome for the high expression of MVP in lung cancer, especially in adenocarcinoma. Therefore, MVP may be associated with the suppression of lung cancer.

NSCLC is thoughted as a group of distinct diseases with genetic and cellular heterogeneity [35]. There are many genetic and epigenetic differences between the main NSCLC histologic subtypes, squamous cell carcinoma and adenocarcinoma. Following the identification of *KRAS* and *BRAF* mutations, epidermal growth factor receptor (*EGFR*) mutations were discovered in patients with lung adenocarcinoma and were associated with response to EGFR inhibitors. Instead, for lung squamous cell carcinoma, genes such as *DDR2*, *FGFR* and genes in the PI3K pathway seem to be more commonly mutated. [35, 36] Difference in the prognosis of MVP expression for adenocarcinoma and squamous cell carcinoma may reflect the complexity of their pathogenesis. For example, STAT3 mediates the oncogenic effects of EGFR kinase domain mutations in human lung adenocarcinoma [37, 38]. The efficacy of EGFR tyrosine kinase inhibitors (TKIs) in EGFR-mutant NSCLC is limited by adaptive activation of STAT3 [39]. The association of high expression of MVP with better clinical outcome in adenocarcinoma may be attributed partly to the suppressive effect of MVP on STAT3 signaling pathway.

However, the anti-tumorigenesis effect of MVP has been challenged in glioblastoma [19] and colon cancer cells [21], in which MVP promotes tumor cell survival and clonogenicity and inhibits cell apoptosis. Furthermore, MVP-mediated selective sorting of tumor suppressor miRNA into exosomes promotes tumor cell growth and colon cancer progression [40]. As an ubiquitously expressed protein, MVP may exert impacts on tumorigenesis in a context-dependent manner. The genetic background, cell linage, and alterations in other pathways may shape the role of MVP in the niche. Indeed, molecules including TGF-β [41], Notch [42], Runx [43], and KLF4 [44], exhibit opposite functions in different cancer settings. The requirement of MVP for the nuclear localization of tumor suppressor PTEN would lead to tumor cell cycle arrest [17]. MVP can also promote the degradation of HIF1α, a known tumor promoting factor, and function as a tumor suppressor in renal adenocarcinoma cells [18]. We demonstrate that MVP may directly inhibit lung cell proliferation and stimulate cell apoptosis. As such, MVP may act as a suppressor for tumorigenesis in lung cancer.

Effect of MVP on signal transduction is also inconsistent as its characteristics in tumorigenesis. MVP has been reported to activate the EGFR/PI3K/AKT signaling pathway in glioblastoma [19] and colon cancer cells [21]. However, MVP also functions as a negative regulator for the growth signaling. For examples, MVP binds with YPEL4 and inhibits YPEL4 catalyzed activation of Elk-1 in the MAPK signaling pathway [45]. Our results reveal that MVP inhibited lung cancer growth by suppression of STAT3 signal pathway, which is regulated by JAK2 and RAF-MEK-ERK pathways. This is consistent with the observation that MVP binds with and inhibits Src kinase activity and, thus, suppresses ERK activation in stomach cancer cells [46]. However, in human airway smooth muscle cells knockdown of MVP induces cell death by inhibiting STAT3 and Akt signaling [47], which is contradictory to our observation in lung tumor cells. Indeed, cancer cells like NSCLC cells are usually endowed with enhanced progrowth signals to satisfy their uncontrolled proliferation [48]. The property of being phosphorylated suggests that MVP be an intrinsic signal transducer [46, 49]. MVP can bind with some signal molecules, such as Src, SHP2, ERK [46, 49], and transcription factors C-FOS and C/EBPβ [50]. Therefore, the mechanisms underlying suppression of STAT3 signaling in lung cancer cells by MVP is warranted further exploration.

## Conclusions

We report here that MVP is associated with a better prognosis of lung adenocarcinoma. MVP may suppress tumor cell growth and facilitate apoptosis of lung cancer cell through inhibition of STAT3 signaling pathway. Our results suggest that MVP act as a suppressor of lung cancer.

## Additional files

Additional file 1: **Figure S1.** The expression of MVP and prognosis in NSCLC. **A-D.** The Kaplan–Meier curves depict the overall survival of NSCLC patients according to their pathology (A-B) and treatment (C-D) (http://kmplot.com/analysis/). **E-G.** The Kaplan–Meier curves depict the overall survival of NSCLC patients according to their pathology in our established cohort. (TIF 1418 kb)

Additional file 2: **Figure S2.** Representative flow cytometry of CD11b^+^ macrophages, Gr-1^+^ neutrophils (A, B), F4/80^+^CD11c^+^ and F4/80^+^CD206^+^ macropahges (C, D) in the transfected LLC cells xenografted tumors in mice. (*n* = 8). (TIF 1507 kb)

Additional file 3: **Figure S3.** MVP knockdown promotes human lung adenocarcinoma SPC-A1 cell growth and inhibits the apoptosis. **A.** Representative western blot of human MVP (hMVP) in the transfected SPC-A1 cells (*n* = 3, ** *P* < 0.01). **B.** Colony formation assays of the transfected SPC-A1 cells. (*n* = 3, ** *P* < 0.01). **C.** CCK8 assays for the proliferation of the transfected SPC-A1 cells. (*n* = 6, ** *P* < 0.01). **D.** Representative flow cytometry of the transfected SPC-A1 cells with PI and Annexin V-647 staining. (*n* = 3, ** *P* < 0.01). (TIF 2959 kb)

## Abbreviations

AKT: AKT serine/threonine kinase
ALK: Anaplastic lymphoma kinase
CCK-8: Cell counting kit-8 assay
CDKs: Cyclin-dependent kinases
DAPI: 4’, 6-diamidino-2-phenylindole
DMEM: Dulbecco’s Modified Eagle Medium
Edu: 5-ethynyl-2’-deoxyuridine
EGF: Epidermal growth factor
EGFR: Epidermal growth factor receptor
Elk-1: ETS transcription factor
EML4: Echinoderm microtubule-associated protein-like 4
ERK: Extracellular regulated MAP kinase
FBS: Fetal bovine serum
HIF1α: Hypoxia-inducible factor 1α
HR: Hazard ratio
IHC: Immunohistochemistry
IRS: Immunoreactivity score
JAK2: Janus kinase 2
KLF4: Kruppel-like factor 4
K-RAS: Kirsten rat sarcoma 2 viral oncogene homolog
LLC: Lewis lung carcinoma
LRP: Lung resistant protein
MAPK: Mitogen activated protein kinase
MEK: MAP kinse-ERK kinase
MET: Mesenchymal-epithelial transition
MVP: Major vault protein
NSCLC: Non-small cell lung cancer
PBS: Phosphate buffer saline
PFA: Paraformaldehyde
PI: Propidium iodide
PI3K: Phosphoinositide 3-kinase
PTEN: Phosphatase and tensin homolog
RAF: Raf oncogene
ROS1: ROS proto-oncogene 1
SHP2: Src homology 2 domain-containing tyrosine phosphatase
STAT3: Signal transducer and activator of transcription 3
TGF-β: Transforming growth factor-β
TKIs: Tyrosine kinase inhibitors
TMA: Tissue microarray
YPEL4: Yippee like protein 4
