# Supplementary figures and images for: Major vault protein suppresses lung cancer cell proliferation by inhibiting STAT3 signaling pathway
Source: BMC Cancer. 2019 May 15;19:454. doi: 10.1186/s12885-019-5665-6 (PMC6521381; doi:10.1186/s12885-019-5665-6)

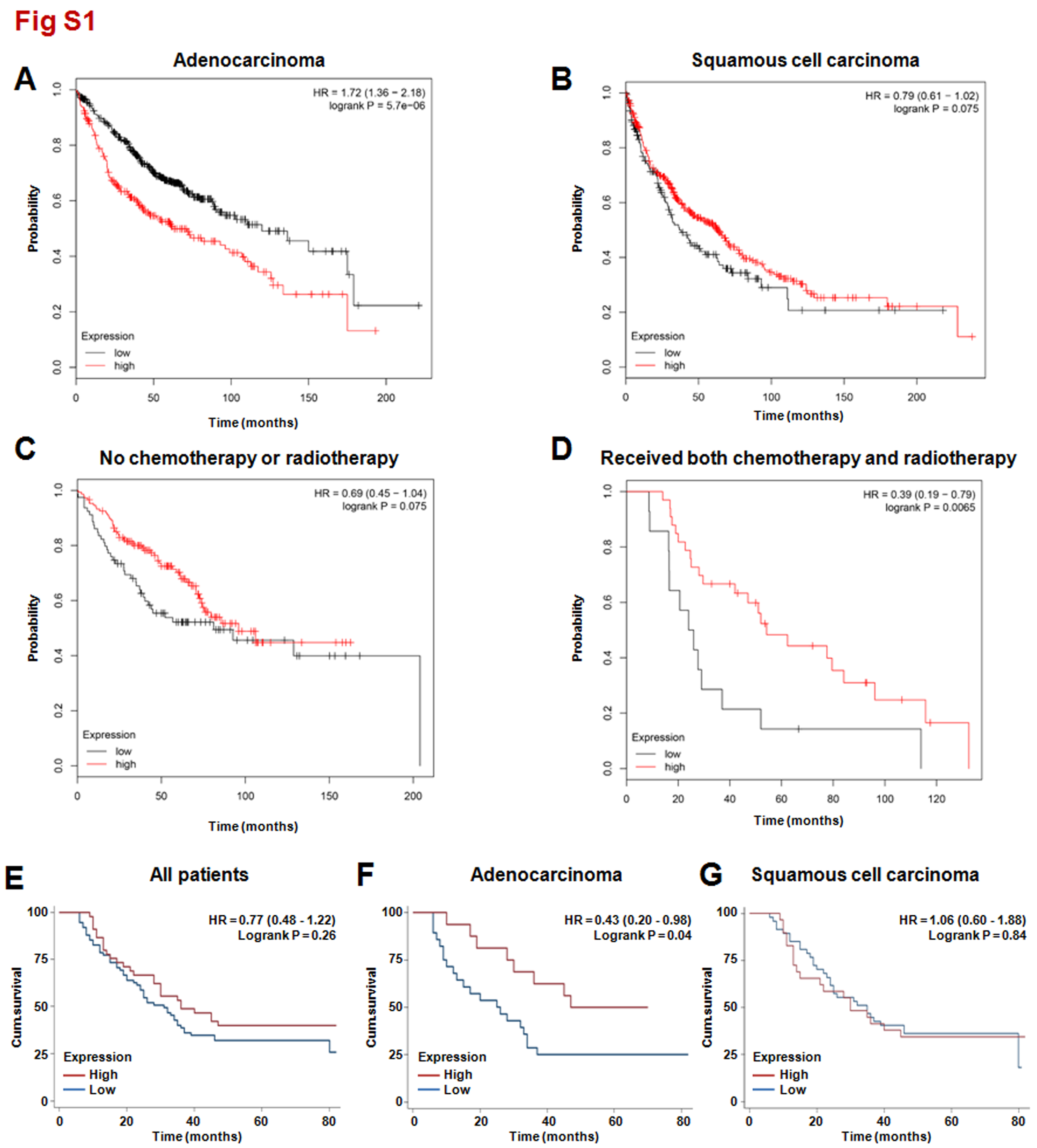

Supplement: Supplementary file 1 — Figure S1. The expression of MVP and prognosis in NSCLC. A-D. The Kaplan–Meier curves depict the overall survival of NSCLC patients according to their pathology (A-B) and treatment (C-D) (http://kmplot.com/analysis/). E-G. The Kaplan–Meier curves depict the overall survival of NSCLC patients according to their pathology in our established cohort. (TIF 1418 kb) [file 12885_2019_5665_MOESM1_ESM.tif]

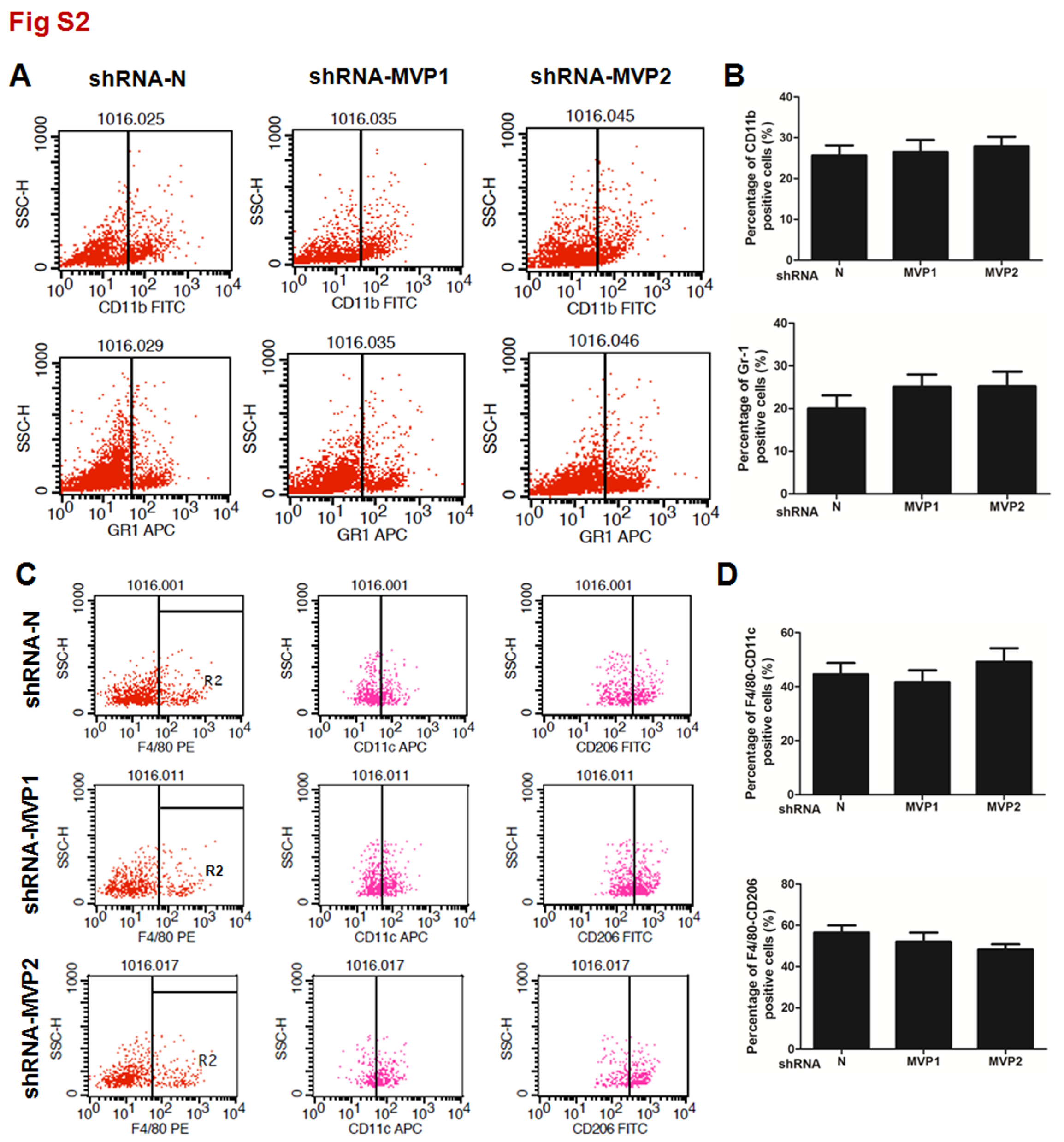

Supplement: Supplementary file 2 — Figure S2. Representative flow cytometry of CD11b+ macrophages, Gr-1+ neutrophils (A, B), F4/80+CD11c+ and F4/80+CD206+ macropahges (C, D) in the transfected LLC cells xenografted tumors in mice. (n = 8). (TIF 1507 kb) [file 12885_2019_5665_MOESM2_ESM.tif]

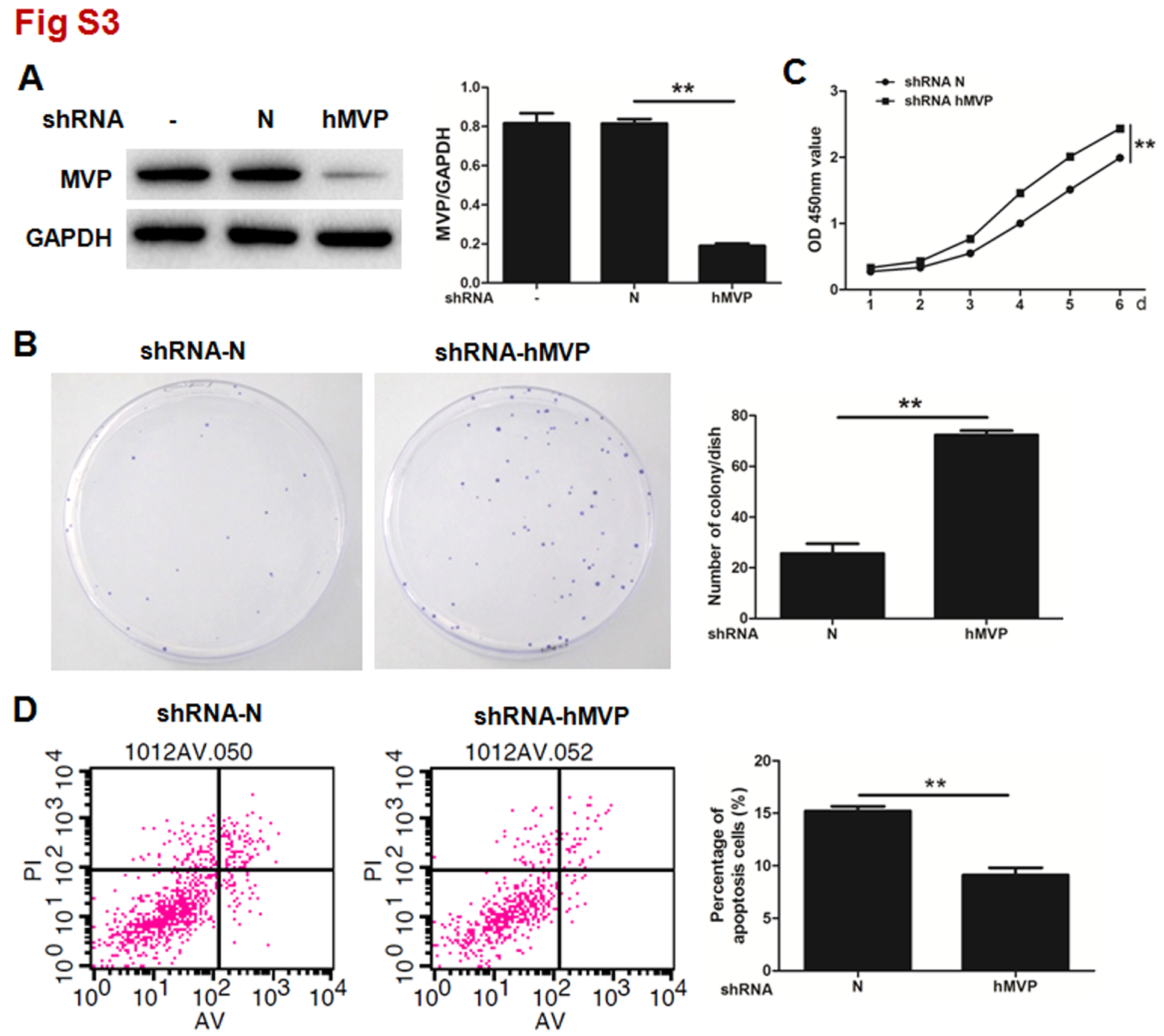

Supplement: Supplementary file 3 — Figure S3. MVP knockdown promotes human lung adenocarcinoma SPC-A1 cell growth and inhibits the apoptosis. A. Representative western blot of human MVP (hMVP) in the transfected SPC-A1 cells (n = 3, ** P < 0.01). B. Colony formation assays of the transfected SPC-A1 cells. (n = 3, ** P < 0.01). C. CCK8 assays for the proliferation of the transfected SPC-A1 cells. (n = 6, ** P < 0.01). D. Representative flow cytometry of the transfected SPC-A1 cells with PI and Annexin V-647 staining. (n = 3, ** P < 0.01). (TIF 2959 kb) [file 12885_2019_5665_MOESM3_ESM.tif]
